# Outcome and toxicity analysis of single dose stereotactic radiosurgery in vestibular schwannoma based on the Koos grading system

**DOI:** 10.1038/s41598-020-66213-4

**Published:** 2020-06-09

**Authors:** Daniel Rueß, Lea Pöhlmann, Stefan Grau, Christina Hamisch, Mauritius Hoevels, Harald Treuer, Christian Baues, Martin Kocher, Maximillian Ruge

**Affiliations:** 1 Department of Stereotaxy and functional Neurosurgery, Centre of Neurosurgery, University Hospital of Cologne, Cologne, Germany; 2 Department of General Neurosurgery, Centre of Neurosurgery, University Hospital of Cologne, Cologne, Germany; 3 Institute of Radiation Oncology, University Hospital of Cologne, Cologne, Germany

**Keywords:** Radiotherapy, Neurology, Surgical oncology

## Abstract

Stereotactic radiosurgery (SRS) has evolved as widely accepted treatment option for small-sized (Koos I up to II) vestibular schwannoma (VS). For larger tumors (prevalent Koos VI), microsurgery or combined treatment strategies are mostly recommended. However, in patients not suited for microsurgery, SRS might also be an alternative to balance tumor control, hearing preservation and adverse effects. The purpose of this analysis was to evaluate the efficacy and toxicity of SRS for VS with regard to different Koos grades. All patients with untreated VS who received SRS at our center were included. Outcome analysis included tumor control, preservation of serviceable hearing based on median pure tone averages (PTA), and procedure-related adverse events rated by the Common Terminology Criteria for Adverse Events (CTCAE; v4.03) classification. In total, 258 patients (median age 58 years, range 21–84) were identified with a mean follow-up of 52 months (range 3–228 months). Mean tumor volume was 1.8 ml (range 0.1–18.5). The mean marginal dose was 12.3 Gy ± 0.6 (range 11–13.5). The cohort was divided into two groups: A (Koos grades I and II, n = 186) and B (Koos grades III and IV, n = 72). The actuarial tumor control rate was 98% after 2 years and 90% after 5 and 10 years. Koos grading did not show a significant impact on tumor control (p = 0.632) or hearing preservation (p = 0.231). After SRS, 18 patients (7%) had new transient or permanent symptoms classified by the CTCAE. The actuarial rate of CTCAE-free survival was not related to Koos grading (p = 0.093). Based on this selected population of Koos grade III and IV VS without or with only mild symptoms from brainstem compression, SRS can be recommended as the primary therapy with the advantage of low morbidity and satisfactory tumor control. The overall hearing preservation rate and toxicity of SRS was influenced by age and cannot be predicted by tumor volume or Koos grading alone.

## Introduction

Due to the widespread availability of magnet resonance tomography imaging (MRI), the incidence of newly diagnosed vestibular schwannoma (VS) has increased over the last 30 years^1^. In general, there are three established management options: (i) microsurgical removal, (ii) radiotherapy (radiosurgery or fractionated radiation therapy) and (iii) “wait and scan“ strategies. Stereotactic radiosurgery (SRS) has evolved as a first-line treatment alternative to surgery since it can achieve tumor control rates between 91–100% in selected patient groups with small, growing VS^2–5^. Due to the lack of alternative grading systems, the decision for treating patients with either surgery or radiosurgery is often based on the Koos grading system. Although this scheme includes a qualitative estimation of both the size and localization of the tumor^6^, it was developed mainly for neurosurgical purposes^7^ and may not be adequate for predicting outcome and toxicity after SRS. The purpose of this study was to evaluate whether the initial Koos grading is a suitable indicator for tumor control, clinical outcome, and toxicity after stereotactic radiosurgery. Therefore, we reviewed sporadic unilateral VS patient cases who underwent SRS with respect to the predictive value of the initial Koos grading alone or in conjunction with other potential predictive factors.

## Methods

### Ethics statement

The Ethic Committee of the University Hospital of Cologne approved the study protocol (Identity: Az 16–476). Due to the retrospective character, the Ethic Committee waived the need for informed consent. All methods were performed in accordance with the relevant guidelines and regulations of the professional code of conduct of the Medical Associations of Nordrhein from 15^th^ of November 2015 (§15, article 1).

### Subjects and populations

In this single center retrospective analysis, we included all patients who received SRS with a radiation dose of less than 14 Gy for unilateral, previously untreated VS. Between 1991 and 2012, patients were treated with a modified linear accelerator (LINAC), and from 2013 onwards patients were treated by robotic radiosurgery using the Cyberknife^R^ system (CK). Baseline data included patient characteristics (age, gender, Koos grade, tumor volume) and relevant radiosurgical parameters (coverage, prescribed dose, maximal dose). Objective pre- and post-treatment hearing impairment was evaluated with tone audiograms. Pure tone averages (PTA) as defined by the WHO^8^ were calculated based on patients’ tone audiograms. The dB values of 500 Hz, 1000 Hz, 2000 Hz and 4000 Hz were summarized and averaged afterwards. Hearing loss up to a PTA level of 50 dB was defined as serviceable hearing, PTA levels between 51 dB and 90 dB as loss of serviceable hearing, and PTA levels of more than 90 dB were categorized as deafness according to the Gardner-Robertson Grades^9^.

Further clinical evaluation was carried out by interviewing individual patients about tinnitus, vertigo, imbalance, and facial motor and sensory function. Any side effects occurring during the follow-up period were rated according to the Common Terminology Criteria for Adverse Events (CTCAE; v4.03, pp 51–55, section “Nervous system disorders”). The adverse event “acoustic nerve disorder NOS” was excluded due to the fact that the patient already had impairments of the cranial nerve (CN) VIII as primary symptoms due to their VS.

### Tumor control

Prior to SRS, tumors were classified according to the Koos grading system^7^. For evaluation of radiological follow-up (FU), contrast-enhanced T1-weighted magnetic resonance images (MRI) were compared with the initial MRI prior to SRS. FU was scheduled at 6 and 12 months after treatment, followed by annual controls. Radiological tumor control was carried out by measuring the largest axial tumor diameter in a.p. and lateral extension on T1-weighted MRIs since this method is traditionally used in numerous retrospective studies^10^. A volumetric FU was not feasible because the majority of the MRIs before 2008 were only available as printed images. Changes in tumor size after SRS were categorized as suggested by Matsuo *et al*.:^3^ 1) enlargement, 2) transient enlargement, 3) stable, and 4) shrinkage. Loss of tumor control was defined as radiological tumor growth with diameters of more than 3 mm according to Hsu *et al*^11^. two or more years after SRS^12^. According to earlier studies^5,13^, clinical tumor control (meaning treatment failure) was defined as freedom from planned or realized re-intervention (e.g. repeated radiosurgery or microsurgery).

### Radiosurgical treatment planning and delivery

Before 1996, the tumor and adjacent critical structures (e.g. brainstem, cerebellum, trigeminal nerve) were outlined by an experienced neurosurgeon on stereotactic planning CT images, although MR imaging was increasingly used for this purpose when available. Since 1996, the tumor was routinely outlined on contrast enhanced, T1-weighted MRI (Phillips, MR-Scanner 1.5 or 3 Tesla), which was obtained prior to SRS and registered to the stereotactic planning CT (1 mm slice thickness, Phillips 8-slice or 16-slice multidetector CT; since 2012 Toshiba 16-slice multidetector CT). Since 2008, a standardized MRI protocol as previously described^4,5^ was used.

In the case of LINAC-based SRS, the patient’s head was immobilized under local anesthesia in a stereotactic frame (Riechert-Mundinger). The SRS planning was carried out using the software STP (STP 3.3 and 3.5, Howmedica Leibinger, Freiburg, Germany). Subsequently, the radiosurgical treatment was performed by using a linear accelerator as previously described^14^. For CK-based SRS, the patient was immobilized on the Cyberknife® treatment table (Accuray, Sunnyvale, California) using a custom-made aquaplast mask. The software Multiplan v4.5 was used for treatment planning. The final irradiation plan was evaluated in an interdisciplinary consensus meeting between the stereotactic neurosurgeon, a radiation oncologist experienced in SRS, and the medical physicist.

### Statistical analysis

Descriptive summaries were prepared for the patients’ demographics. To facilitate the comparison, Koos grades I and II were aggregated into “group A” and grades III and IV into “group B”. An unpaired t-test was used to compare metric features of both groups. Categorical features were compared using chi-square test. The Wilcoxon matched pairs signed rank test was used to compare tumor size before SRS and at last follow-up. An univariate analysis (logrank test) was used to compare subgroups of variables. A *p-*value of <0.05 was considered statistically significant. Additionally, a multivariate analysis (Cox proportional hazards model) with backward selection and a removal level of p > 0.05 was used to evaluate the influence on radiological and clinical tumor control, serviceable hearing preservation and CTCAE-toxicity-free rates. The model includes the following variables: age, gender, tumor volume (TV), Koos grades, co-morbidities, radiation dose to the tumor margin, and coverage. The statistical analysis was performed using the software Graphpad PRZM 8.0 and SPSS 25.0.

### Ethical approval

All procedures performed in studies involving human participants were in accordance with the ethical standards of the institutional and/or national research committee and with the 1964 Helsinki declaration and its later amendments or comparable ethical standards. Due to the retrospective character of this study formal consent was not required. The Ethic Committee of the University Hospital of Cologne approved the study protocol (Identity: Az 16–476) and waived the need for informed consent. All methods were performed in accordance with the relevant guidelines and regulations of the professional code of conduct of the Medical Associations of Nordrhein from 15^th^ of November 2015 (§15, article 1).

(see methods section).

## Results

### Patient collective and tumor characteristics

A total of 258 patients (female/male = 129/129) with a median age of 58 years (range 21–84 years) were analyzed (Table 1). Overall median follow-up (FU) was 35 months (range 3–228 months) and mean follow-up was 52 months. About one third (n = 81, 31.4%) of the patients had a FU period extending over 5 years. LINAC SRS was performed between 1991–2012 in 207 patients. Since 2013, 51 patients were treated with the Cyberknife®. There was a significant difference (p < 0.0001) between FU length of LINAC patients (median FU 60 months, range 2–228 months) and Cyberknife® patients (median FU 20.8 months, range, 4–61 months).

**Table 1:** Clinical characteristics and treatment parameters of patients.

n = 258	Group A (Koos I/II) n = 186	Group B (Koss III/IV) n = 72	p value
Patient characteristics			
**m: f**	**93:93**	**36:36**	***0.55***
**Median age (years)**	**58 (*21–80*)**	**63 (*25–84*)**	***0.11***
**Mean tumor vol. (ml)**	0.6* ± 0.5 (*0.1–2.7*) Koos I: 0.2 ± 0.13 (*0.1–0.68*) Koos II: 0.93 ± 0.5 (*0.24–2.7*)	4.3* ± 2.5 (*1.4–18.5)* Koos III: 3.1 ± 1.1 (*1.4–5.8*) Koos IV: 5.5 ± 3 *(1.6–18.5)*	**<*0.0001***
**Median FU (months)**	**34 (*3–228*)**	**39 (*2–224*)**	***0.24***
**Co-morbidities (%)**	**94 (50.5%)**	**44 (61%)**	***0.061***
**Malignancy (%)**	6* (3.2%)	9* (12.5%)	***0.007***
**Initial symptoms and signs**			
**Hearing disturbance (%)**	**153 (82.3%)**	**64 (88.9%)**	***0.131***
- serviceable	141* (75.8%)	39* (54.2%)	***0.001***
- non-serviceable	34* (18.3%)	26* (36.1%)	***0.002***
- Hearing loss (%)	**11 (5.9%)**	**7 (9.7%)**	***0.207***
**Vertigo (%)**	**86 (46.2%)**	**35 (48.6%)**	***0.137***
**Imbalance (%)**	**45 (24.2%)**	**23 (32%)**	***0.134***
**Tinnitus (%)**	57* (30.6%)	8* (11.1%)	***0.001***
**CN V impairment (%)**	2* (1.1%)	8* (11.1%)	***0.001***
**CN VII impairment (%)**	**8 (4.3%)**	**2 (2.8%)**	***0.438***
**Radiation parameters**			
**LINAC (1991–2012)**	**147**	**60**	***0.437***
**CK (2013–2015)**	**39**	**12**	***0.277***
**Mean marginal dose (Gy)**	**12.4** ± **0.5 (*11–13.5*)**	**12.2** ± **0.5 (*11–13*)**	***0.17***
**Isodose prescription (%)**	74* ± 9.5 (*40–86*)	70* ± 17.5 (*33–85*)	***<0.001***
**Coverage (%)**	**98.5** ± **2.2 (*range: 89–100*)**	**99.1** ± **1.8 (*range:*** **93–99.9)**	***0.52***

There were statistically significant differences (p < 0.05) between groups if marked with *.

The mean marginal dose delivered to all tumors independently of the radiation system was 12.4 ± 0.8 Gy (range, 11.0–20.0 Gy). The prescription isodose was 69 ± 12.5% (range 33–86%). According to the Koos classification, 45 tumors were intrameatal tumors (Koos I; 17.4%), 141 were intra- and extrameatal tumors (Koos II; 54.6%), 36 were intra- and extrameatal tumors with contact to the brainstem and exceeding 2 cm in diameter (Koos III; 14%), and 36 were intra- and extrameatal tumors with compression of the brainstem (Koos IV; 14%). The cohort was split into two groups: A (Koos grades I and II) and B (Koos grades III and IV) (Table 1). Besides cranial nerve V impairment and tinnitus, the initial symptoms prior to therapy were almost similarly distributed in both groups.

The average tumor volume (TV) differed significantly (p < 0.0001) between the groups (A: 0.8 ± 0.5 ml, range: 0.1–2.7 ml, B: 4.3 ± 2.5 ml, range: 1.4–18.5 ml), but the distributions clearly overlapped (Table 1). Group B showed a higher amount of malignant co-morbidities (Table 1, p = 0.007).

### Tumor control

**Figure 1:**
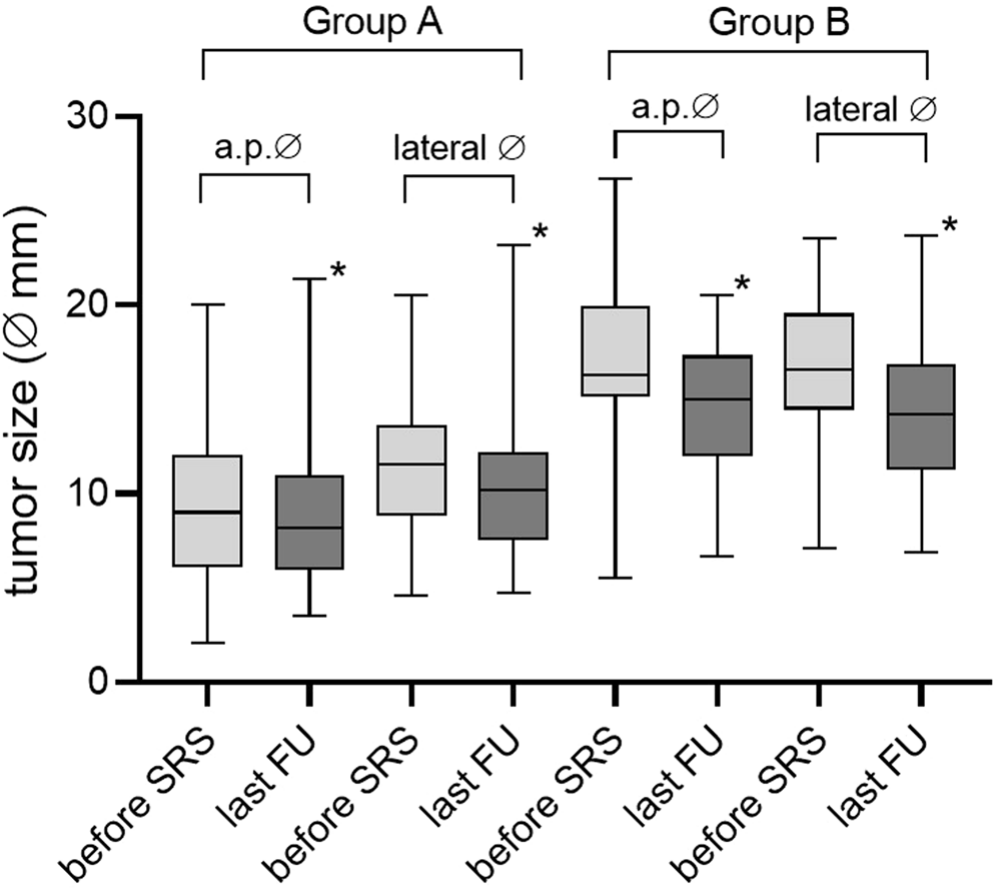
Comparison of tumor size in a.p. and lateral diameter before and after SRS for groups of Koos grades. In both groups, tumors decreased significantly in size at last FU (*p = 0.01, group A: Koos grades I and II, group B: Koos grades III and IV).

Tumor size was monitored in all patients by follow-up MRI. At the last follow up, 24% (n = 62) of the tumors were categorized as shrinkage, 65% (n = 167) as stable and 11% (n = 29) as enlarged. Transient enlargement was found in 20.1% (n = 52). Overall tumor size (a.p. and lateral diameters) significantly decreased in all groups and dimensions (Fig. 1). In detail, measurement of the tumor size in group A revealed an average a.p. diameter of 9.5 ± 3.7 mm (range 2.1–20.0 mm) before SRS and 8.9 ± 3.8 mm (range 3.5–21.4 mm) at the last FU. The lateral dimension before SRS was 11.6 ± 3.4 mm (range 4.6–20.5 mm) and 10.2 ± 3.5 mm (range 4.7–23.2 mm) at the last FU. In group B, the average a.p. diameter was 17.0 ± 5 mm (range 5.5–28.4) before SRS and 14.1 ± 4 mm (range 6.0–20.5 mm) at the last FU, and the lateral dimension before SRS was 16.7 ± 3.6 mm (range 7.1–23.5 mm) and 14.3 ± 3.8 mm (range 6.9–23.7 mm) at the last FU (Fig. 1).

**Figure 2:**
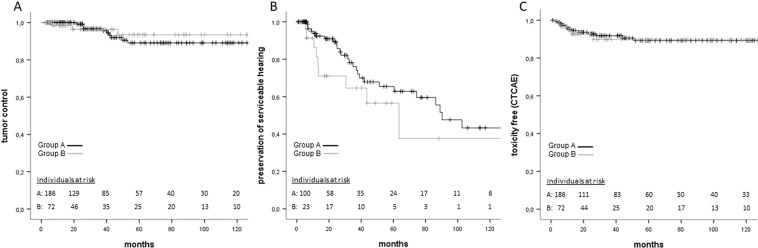
(A) Kaplan-Meier analysis of actuarial tumor control after SRS of unilateral VS for group A (Koos grade I/II) and B (Koos grades III/IV). There was no significant difference (logrank, p = 0.632) between either group. (**B**) Kaplan-Meier analysis of hearing preservation rate between group A and B. Log rank test and multivariate analysis revealed no statistically significant impact (logrank, p = 0.231) on hearing preservation rate. (**C**) Toxicity free survival in terms of permanent CTCAE showed no statistically significant difference in a logrank test (p = 0.93).

Overall loss of tumor control was noted in 13 patients (5%). Kaplan-Meier analysis revealed an actuarial tumor control rate of 98% after 2 years and 90% each after 5 and 10 years. There were no significant differences (p > 0.632) between subgroups of Koos grades (Fig. 2A) or for any other factors tested (Table 2.).

**Table 2:** Prognostic factors of VS treated with SRS.

Analysis for TC				
Log rank		Multivariate		
*Factors*	*P value*	*P value*	*HR*	*95% CI*
Age	0.131	0.442	0.96	(0.88–1.05)
Gender	0.111	0.995	0.99	(0.18–5.4)
Co-morbidities	0.462	0.932	1.1	(0.1–11.4)
Tumor volume	0.912	0.53	0.65	(0.17–2.5)
Gray	0.866	0.94	0.93	(0.1–6.7)
Coverage	0.526	0.63	0.918	(0.64–1.3)
Koos Grade	0.632	0.765	0.56	(0.01–23.08)
**Analysis for hearing preservation**				
**Log Rank**		**Multivariate**		
***Factors***	***P value***	***P value***	***HR***	***95% CI***
Age	0.096	0.017*	1.05	(1.01–1.1)
Gender	0.954	0.525	1.46	(0.45–4.7)
Co-morbidities	0.678	0.26	2.4	(0.51–11.43)
Tumor volume	0.591	0.89	1.04	(0.52–2.08)
Gray	0.128	0.363	1.87	(0.48–7.3)
Coverage	0.176	0.23	1.34	(0.82–2.19)
Koos Grade	0.231	0.47	0.49	(0.07–3.3)
**Analysis freedom of toxicity (CTCAE)**				
**Log Rank**		**Multivariate**		
***Factors***	***P value***	***P value***	***HR***	***95% CI***
Age	0.763	0.181	0.961	(0.9–1.02)
Gender	0.426	0.725	0.782	(0.2–3.01)
Co-morbidities	0.057	0.326	0.469	(0.1–2.1)
Tumor volume	0.837	0.341	1.48	(0.65–3.3)
Gray	0.061	0.087	4.87	(0.8–29.66)
Coverage	0.411	0.958	0.989	(0.65–1.5)
Koos Grade	0.93	0.401	3.31	(0.2–54.17)

*P value *<*0.05 is considered significant.

### Preservation of serviceable hearing

The most frequent symptom prior to SRS was hearing disturbance (group A: n = 153 (82.3%), group B: 64 (88.9%)). In groups A and B, 141/186 (75.8%) and 39/72 (54.2%) patients had serviceable hearing prior to SRS. In 126 patients (group A: n = 100, group B: n = 23), pure tone audiograms were available which were used to objectify hearing levels (PTA) prior to and after SRS until the last FU. In groups A and B, 70% (n = 71/100) and 60% (n = 14/23) retained serviceable hearing at the last FU. The Kaplan-Meier analysis estimated an overall preservation rate for subjective serviceable hearing of 84% after 2 years and 61% after 5 years. However, Koos grading did not significantly impact (p = 0.213) preservation of serviceable hearing (Fig. 2B). Age had a significant impact on preservation rate of serviceable hearing (p = 0.017, 95%-CI 1.01–1.1, HR 1.05, Table 2).

### Toxicity and adverse events

Overall, new symptoms classified as adverse events according to the CTCAE occurred in 36 (14%) out of 258 patients. Permanent adverse events lasting until the last FU were present in 18 patients (7%, Table 3) of whom 13 patients were in group A and 5 patients in group B. Notably, the occurrence of any CTCAE-classified permanent adverse events was not related to Koos grading (p = 0.93, Fig. 2C). However, multivariate analysis did not reveal any significant impact on toxicity (CTCAE) free survival rate (Table 2).

**Table 3:** Overview of permanent side-effects after SRS using CTCAE criteria.

CTCAE Grading	Group A			Group B		
	Grade 1	Grade 2	Grade 3	Grade 1	Grade 2	Grade 3
Imbalance, n = 5	2	1	–	1	1	–
CN VII disorder, n = 9	3	3	1	1	1	–
CN V disorder, n = 8	4	–	–	3	1	–
Hydrocephalus, n = 2	–	1	–	–	1	–
Endema, n = 1	–	–	–	–	1	–

A total of 20 patients (7.5%) reported permanent complaints after SRS. In some cases multiple symptoms occurred. In our series CTCAE criteria were grade 1 (mild symptoms, asymptomatic or mild symptoms without impact on daily life), grade 2 (moderate, minimal, local or noninvasive intervention indicated; limiting age-appropriate instrumental activities of daily life (ADL)), and grade 3 (severe or medically significant, but not immediately life-threatening; hospitalization or prolongation of hospitalization indicated; disabling; limiting self care ADL). We did not find any adverse events matching grade 4 (life-threatening consequences; urgent intervention indicated) or grade 5 (death related to AE).

## Discussion

Among other factors, the decision about treatment for VS is mainly based on tumor size, and in this regard, Koos grading is generally viewed as a useful indicator. Particularly for VS with higher Koos grades (≥ III), microsurgery is suggested as standard care, either used as stand-alone^15,16^ or as combined treatment^17,18^.

### Surgery

Generally speaking, the goal of surgery is tumor resection to the maximal possible extent with preservation of neurologic functions. In the case of larger VS with compression of brainstem and critical structures, relief of symptoms and reduction of pressure is required. Consequently, SRS was considered as a contraindication for VS with Koos grades III and IV^2,19,20^, especially due to the well-known phenomenon of pseudoprogression, which can occur after SRS and may lead to new disorders and side-effects. Therefore, it was argued that the risk of SRS might increase with higher Koos grades; but is it not likely that the risk also increases in higher Koos grade tumors treated by microsurgery?

In stand-alone surgery of larger VS, the rates of postoperative facial paralysis (House and Brackmann grade IV-V) at the last FU reach 18–25%^21,22^. Whether tumor size is related to postoperative rates of facial paralysis is still a matter of debate. Whereas Bloch *et al*^23^. did not find a significant correlation between tumor size and CN VII palsy, a review by Shugrue *et al*^24^. comprising> 30,000 patients revealed a significantly higher risk for vascular injuries, neurological deficits, and infections in tumors exceeding 25 mm. Apart from neurological deficits, the rate of hearing preservations seems to be influenced by tumor size. Gross total tumor resections of> 1000 VS exceeding 30 ×20 mm in size resulting in a poor hearing preservation rate of 24.2% after 5 years were reported by Huang *et al*^21^. Additionally, a large review by Shugrue *et al*^25^. identified a tumor size of> 15 mm as a risk factor for hearing loss. Thus, current guidelines^26^ state that tumor size is among the most reliable prognostic factors for hearing preservation and CN VII function following microsurgery of VS.

In summary, microsurgery of large tumors carries a substantial risk for side-effects. Hence, there may be cases of higher Koos grade tumors with an inferior risk-benefit ratio for microsurgery compared to primary SRS. Especially patients with Koos grades III-IV who present with low morbidity and mild symptoms might be candidates for SRS. In addition, patients who are not suited for surgery due to co-morbidity, age, and individual preferences might also benefit from stand-alone SRS.

### Radiosurgery

Wolbers *et al*^6^. analyzed two prospective and four retrospective controlled trials which compared patients’ outcome after surgery or SRS of VS. They concluded that tumors up to 30 mm in diameter (equaling Koos grades I – III) benefitted from SRS instead of microsurgery. In the case of tumors exceeding 30 mm in diameter (equaling Koos grade IV), the authors stress the lack of data/studies. So far, it is still under debate whether SRS of VS with higher Koos grades leads to a higher toxicity or inferior tumor control rates. Thus, our study is one of the first that compares these subgroups with respect to tumor control, hearing preservation, and CN toxicity.

According to recent publications^27–33^, our study is the first that comprises LINAC and Cyberknife® data (Table 4). Tumor control rates in our series, in those from the literature and in other SRS series with smaller VS^2,5,34–36^, varied between 87–100%. The largest study of Cyberknife® SRS of VS so far was published by Windisch *et al*.^37^. with> 1000 patients and revealed an overall tumor control rate of 92% after five years. Tumor volume was a significant predictor of local control. Larger volumes (>0.5 ml) showed worse control in both the Cox proportional hazards model and the log-rank test. Regarding this study it has to be mentioned that tumor control was defined as increased size in two consecutive FU, which differs from other studies mentioned in Table 4. Since Windisch *et al*.^37^ did not measure tumor size or volume systematically in FU and Koos grades were not defined their findings remain vague. In our study, successful tumor control after SRS does not depend on tumor volume and/or Koos grade. Our data demonstrate tumor control rates and toxicity levels within range of previously published studies (Table 4). Since tumor size did not affect the rate of tumor control or the incidence of side-effects, the proposed dogma of tumor size being the most relevant limitation for SRS may not be valid in all cases.

**Table 4:** Characteristics of preexisting retrospective single-center series dealing with the radiosurgical treatment of large VS.

Authors, Center & Year	n	SRS System	Median FU (months)	Median tumor volume (ml)	5-y-clinical tumor control	5-y-Hearing preservation rate	5-y-toxicity (CTCAE) free survival rate	Cranial nerve impairment (%)
Chung *et al*., Taipei, 2010^28^	**21**	GK	**53**	**17.3** (12.7–25.2)	**93.8%**	N/a	N/a	**0% Imbalance 23% (n** = **5)**
Yang *et al*., Pittsburgh, 2011^33^	**65**	GK	**36**	**9** (5–22)	**87%**	**82%**	N/a	**CN V 6% CN VII 2%**
Milligan *et al*., Minnesota, 2012^32^	**22**	GK	**66**	**5.4** (5–19)	**91%**	**28%**	N/a	**CN V 14% CN VII 14%**
Bailo *et al*., Milano, 2016^27^	**59**	GK	**36**	**5.9** (2.5–14.9)	**97.9**	**28%**	N/a	**CN V 6.1% CN VII 5.8%**
Ioro-Morin *et al*., Quebec, 2016^30^	**68**	GK	**47**	**7.4** (4–19)	**92%**	**49%**	N/a	**CN V 15%**
Lefranc *et al*., Marseille, 2018^31^	**86**	GK	**72**	**4.4** (1.3–8.7)	**90.7%**	**65%**	N/a	**0%**
Our series (VS of Koos III and IV)	**72**	**LINAC 60 CK 12**	**39**	**4.3** (1.4–18.5)	**93.4%**	**56.5%**	**88%**	**CN VII 2.7% CN V 5.5%**

Hearing is influenced by multiple factors (age, cochlea radiation dose, pre-therapeutic hearing class) making the interpretation of a SRS as an isolated factor difficult. This multifactorial complexity is reflected by the heterogeneity of published results reporting preservation rates between 28 and 82% (Table 4). Following current literature, tumor size definitely has an impact on hearing preservation during surgery and smaller tumors are also linked to a higher chance of preservation after SRS^5,38,39^. In contrast to that, we could not confirm these observations in our study. One reason for this might be the high amount of non-serviceable hearing prior to SRS in the cohort with Koos grade III/IV tumors. We found age to be the only significant influence in multivariate analysis. A worse hearing outcome with increasing age after SRS is discussed controversially. Some studies have suggested that advanced age results in poorer hearing outcomes^40,41^, whereas others did not^42^. However, apart from the influence of radiotoxicity, whether there is an increasing influence on presbyacusis is still an open question.

An important topic is the toxicity of SRS treatment. This study is the first to evaluate the actuarial toxicity-free rate after SRS of VS in terms of CTCAE classification and used it to differentiate between events that occurred permanent and temporarily. In recent literature (Table 4), only studies reporting the crude rate of e.g. cranial nerve impairment (CN VII and V) were found. In series with smaller VS^4,43–45^, the rate of CN impairment ranged from 0–4%, while it increased up to 14% in high-volume tumors (Table 3). Although these results suggest that SRS leads to an increased toxicity in tumors with higher volumes, we could not confirm these findings in logrank tests nor in multivariate cox regression analysis. One reason for this is the homogeneous application of the radiation dose. At higher radiation doses (> 14 Gy), as administered especially in the 1990s, the toxic side-effects were significantly higher^46,47^. Additionally, pseudo progression, which can lead to early adverse events and/or early loss of clinical tumor control, could be kept low especially in tumors with higher volumes. Thus, these high radiation doses should no longer be used in current treatment of VS. In order to make our results comparable with the modern series of SRS treated VS, we excluded all patients in our analysis with radiation doses equal to, or higher than 14 Gy.

### Usefulness of Koos grading system for treatment decision

Whether Koos grading alone provides enough information about the tumor stage is questionable. As expected, in our study and in other systematic volumetric analyses of VS^48^ an overlap between the Koos grades and tumor volume is observed. A controversial finding in our study and in others^31,48^ is that tumor volume does not always correlate with Koos grading. For instance, some Koos grade IV tumors with an elliptic shape may have volumes of less than 2 ml, whereas Koos grade II tumors with a spherical shape could exceed volumes of 2 ml. Additionally, elderly patients facing brain atrophy might have a wider cerebellopontine angle than younger patients, and can probably tolerate higher tumor volumes. This might in part explain why the Koos class did not correlate with outcome parameters in our study. So one might question whether Koos grading alone is suitable for treatment decisions, or whether more comprehensive classification for VS patients including Koos grading, tumor volume, age, clinical condition and pretreatment might facilitate decision making for VS.

## Limitations of the study

Due to the retrospective nature, follow-up times are somewhat limited in our study. There are multiple reasons for this, e.g. lack of patient compliance, long travelling distance between the patient’s place of residence and the treatment site, or changes in the place of residence that may prevent patients from returning to the referring hospital. Therefore, comparison of our data with recent publications (Table 4) is partly limited. In the literature, a five-year interval is mostly given as an example. In our study, the median observation interval for group B is slightly more than three years. Another limitation is the assessment of hearing preservation, which could only be performed in about 70% of the collective. Based on these limitations our results may be overestimated; but on the other hand some individuals had very long FUs of nearly 20 years. Additionally, hearing preservation was objectively analyzed and did not rely on the subjective perception of the individual patient. Furthermore, whether the SRS system in use plays a role remains unclear due to the significantly lower FU length of Cyberknife® treated patients.

However, these limitations and heterogeneity of the cohort may reflect daily practice best.

## Conclusion

Based on this selected population of Koos grade III and IV VS with mild symptoms and without symptoms from brainstem compression, SRS can be recommended as the primary therapy, with the advantage of low morbidity and satisfactory tumor control. The overall hearing preservation rate and toxicity of SRS are influenced by multiple factors, and cannot be predicted by tumor volume or Koos grading alone. At least with regard to SRS, Koos classification alone is not a suitable tool for directing therapy decisions.
